# Novel mouse model of steroid-resistant allergic rhinitis by repeated intranasal administration of OVA

**DOI:** 10.1186/1939-4551-8-S1-A30

**Published:** 2015-04-08

**Authors:** Kazunari Izumi, Hideki Kawasome, Atsushi Azuma, Kenjiro Sasaki, Masayuki Kamada, Kazushi Sakurai

**Affiliations:** 1 Otsuka Pharmaceutical Co., Ltd, Japan

## Background

Allergic rhinitis (AR) is one of uncontrollable inflammation diseases by a corticosteroid administration. Thus an ideal AR animal model had been required in this research field in order to develop a new effective remedy. We report our establishment of the novel AR animal model resistant to nasal corticosteroid.

## Methods

BALB/c mice were sensitized by intraperitoneal injection of ovalbumin (OVA) /ALUM. Two weeks after the sensitization, the mice were administrated OVA intranasally for five times a week (*One-week-challenge arm*), or five days in the first week followed by three times a week for every two days from the second to the fifth week (*Five-week-challenge arm*). In the both arms, the day before the first challenge, the mice were randomly divided into four groups (n=8): negative control (NC), vehicle control (VC), mometasone furoate (MF), and dexamethasone (DEX). For the NC groups, sensitized mice were intranasally treated with saline instead of OVA. In the MF groups, 5 μg/10 μL of MF was administrated intranasally 30 minutes before the last OVA challenge. In the DEX groups, 1 mg/kg of DEX was administrated orally 1 hour before the last OVA challenge. The nasal congestion in early phase and late phase responses were measured by two-chambered, double-flow plethysmograph system as specific air way resistance (sRAW) 10 minutes and 3 hours after the last OVA challenge, respectively. The mice were sacrificed by CO_2_ gas inhalation and the nasal paraffin sections were made for histological evaluation.

## Results

In the *One-week-challenge arm*, the sRAW in MF and DEX group were lower than VC group both in the early (inhibition rates: MF 73%, DEX 94%) and the late phase responses (MF 76%, DEX 84% inhibition). In the *Five-weeks-challenge arm*, MF and DEX reduced the nasal congestion in the early phase (MF 42%, DEX 58% inhibition), however, the inhibition rate of the late phase was significantly decreased in the MF group (MF 28%, DEX 50% inhibition). The epithelial hyperplasia and the inflammatory cells infiltration were observed in the nasal mucosa, and these grades were more remarkable for *Five-week-challenge arm* than *One-week-challenge arm.*

## Conclusions

After five weeks OVA challenge, the nasal congestion remained partially even after the nasal corticosteroid treatment. This repeated OVA administration method would provide a quite useful novel disease model to elucidate the mechanisms of nasal steroid-resistant-AR and develop a new remedy.

